# Long-read RNA-sequencing reveals transcript-specific regulation in human-derived cortical neurons

**DOI:** 10.1098/rsob.250200

**Published:** 2025-07-30

**Authors:** Jishu Xu, Michaela Hörner, Elena Buena Atienza, Kalaivani Manibarathi, Maike Nagel, Stefan Hauser, Jakob Admard, Nicolas Casadei, Stephan Ossowski, Rebecca Schuele

**Affiliations:** ^1^ Centre for Neurology and Hertie Institute for Clinical Brain Research, University of Tübingen, Tübingen, Germany; ^2^ Institute of Medical Genetics and Applied Genomics, University of Tübingen, Tübingen, Germany; ^3^ Graduate School of Cellular and Molecular Neuroscience, University of Tübingen, Tübingen, Germany; ^4^ Division of Neurodegenerative Diseases and Movement Disorders, Department of Neurology, Heidelberg University Hospital and Faculty of Medicine, Heidelberg, Germany; ^5^ NGS Competence Center Tübingen (NCCT), University of Tübingen, Tübingen, Germany; ^6^ German Centre for Neurodegenerative Diseases (DZNE), Tübingen, Germany; ^7^ Interdisciplinary Center for Neurosciences (IZN), Heidelberg, Germany; ^8^ Faculty of Biosciences, University of Heidelberg, Heidelberg, Germany; ^9^ Institute for Bioinformatics and Medical Informatics (IBMI), University of Tübingen, Tübingen, Germany

**Keywords:** long-read RNA-sequencing, transcriptomics, transcript usage, alternative splicing, human-derived cortical neurons, induced pluripotent stem cells

## Abstract

Long-read RNA sequencing has transformed transcriptome analysis by enabling comprehensive mapping of full-length transcripts, providing an unprecedented resolution of transcript diversity, alternative splicing and transcript-specific regulation. In this study, we employed nanopore long-read RNA sequencing to profile the transcriptomes of three cell types commonly used to model brain disorders, human fibroblasts, induced pluripotent stem cells and stem cell-derived cortical neurons, identifying extensive transcript diversity with 15 072 transcripts in stem cell-derived cortical neurons, 13 048 in fibroblasts and 12 759 in induced pluripotent stem cells. Our analyses uncovered 35 519 differential transcript expression events and 5135 differential transcript usage events, underscoring the complexity of transcriptomic regulation across these cell types. Importantly, by integrating differential transcript expression and usage analyses, we gained deeper insights into transcript dynamics that are not captured by gene-level expression analysis alone. Differential transcript usage analysis highlighted transcript-specific changes in disease-relevant genes such as *APP*, *KIF2A* and *BSCL2*, associated with Alzheimer’s disease, neuronal migration disorders and degenerative axonopathies, respectively. This added resolution emphasizes the significance of transcript-level variations that often remain hidden in traditional differential gene expression analyses. Overall, our work provides a framework for understanding transcript diversity in both pluripotent and specialized cell types, which can be used to investigate transcriptomic changes in disease states in future work. Additionally, this study underscores the utility of differential transcript usage analysis in advancing our understanding of neurodevelopmental and neurodegenerative diseases, paving the way for identifying transcript-specific therapeutic targets.

**Figure:**
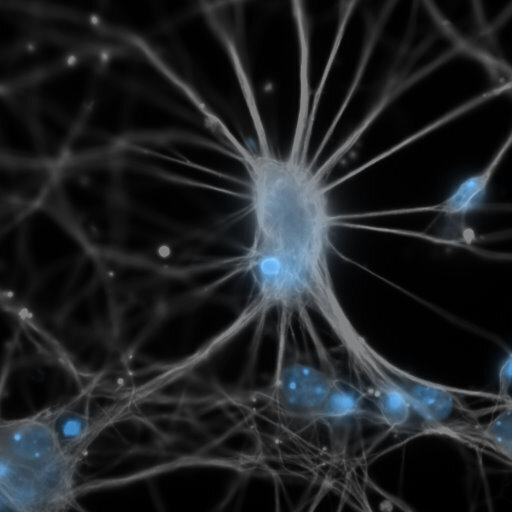

## 1. Introduction

Cellular differentiation is a complex process where cells transition from a pluripotent state to specialized mature phenotypes. This developmental continuum, from pluripotent stem cells to differentiated cell types like fibroblasts or neurons, is critical for understanding developmental biology, disease mechanisms and providing therapeutic innovations [1–3]. Likewise, in translational research, patient-derived fibroblasts are a more easily accessible cell source that can be reprogrammed into induced pluripotent stem cells (iPSC) and further differentiated into otherwise inaccessible cell types—opening new avenues for disease modelling and therapeutic discovery. Robust cell systems have been developed to generate various cell types *in vitro*, including different types of neurons [4–8].

In the study of neurodevelopmental and neurodegenerative diseases, human fibroblasts are widely used as an accessible and clinically relevant somatic cell type, particularly when patient-derived neuronal tissue is unavailable. Hence, fibroblasts are often used as a proxy to study neuronal pathophysiology due to their availability and ease of handling. Additionally, fibroblasts can be reprogrammed into iPSC, which can subsequently be differentiated into various neural lineages, including cortical neurons [9]. This fibroblast-to-iPSC-to-iPSC-derived cortical neuron (iCN) model has been extensively employed to investigate transcriptomic reprogramming and neuronal pathophysiology, especially in patient-derived disease models [9–12]. Despite their widespread use, our understanding of the differences between fibroblasts and neuronal cell types in terms of transcript regulation remains limited. While other ectoderm-derived lineages (e.g. surface ectoderm or ectomesenchyme) may offer developmental context [13,14], our focus is on capturing transcriptomic transitions across the full reprogramming and differentiation trajectory. Moreover, isoform-level comparisons between fibroblasts and iPSC-derived neurons remain underexplored, and our study aims to fill this gap using long-read (LR) RNA sequencing.

Transcriptome studies in most neuronal models have been limited to gene expression studies, which often provide only broad regulatory patterns. These analyses tend to overlook the complexity introduced by alternative splicing (AS)—a process that affects nearly 95% of human pre-mRNAs [15,16]. AS facilitates the generation of diverse transcript variants, enabling fine-tuned regulation of gene expression across tissues and developmental stages [17]. The regulation of AS involves a complex network in which RNA-binding proteins (RBPs) act as cis-regulators by directly interacting with intronic and exonic splice enhancer and silencer sequences. AS is also controlled by trans-regulators, such as transcription factors (TFs), which modulate the transcriptional elongation rate and modify chromatin structure [6,18–23]. A comprehensive review highlights that AS is often coordinated with transcription and regulated by chromatin accessibility and epigenetic modifications, linking splicing outcomes to RNA polymerase II dynamics and histone marks [24]. AS is particularly prevalent in the vertebrate brain, where it plays an essential role in key processes such as neurogenesis, synaptogenesis, axon guidance and neural plasticity [20,25–31]. Quantifying these splice variants can reveal specific patterns associated with different cellular conditions, like neuronal differentiation, or disease states, like Alzheimer’s disease (AD) and other tauopathies [32–36].

While differential gene expression (DGE) studies have been informative, growing evidence highlights the importance of differential transcript expression (DTE) and differential transcript usage (DTU) in decoding complex gene regulation. Computational tools such as SUPPA2 and DEXseq facilitate accurate quantification of AS events from transcript abundance estimates, enabling scalable splicing analysis across multiple conditions [37,38]. These approaches leverage fast and efficient transcript quantification techniques to dissect transcript-level regulation, making them especially suitable for cell differentiation and disease models where AS plays a critical regulatory role.

Importantly, transcript-level analyses have been shown to improve both sensitivity and interpretability of RNA sequencing (RNA-seq) data. For example, Soneson *et al*. [39] demonstrated that incorporating transcript-level abundance estimates enhances detection power in gene-level differential expression workflows [39]. Moreover, transcript- and isoform-level analyses have revealed prognostic signatures and cancer-specific splicing networks that remain undetectable when relying solely on gene-level data [40,41]. By mapping the full landscape of AS and transcript diversity, LR sequencing offers unprecedented resolution in understanding gene regulation and its implications for human health and disease.

Although traditional short-read RNA-seq has provided invaluable insights into the transcriptome, its limitations—such as an inability to accurately resolve complex transcript structures and quantify quantifying AS events—are well documented [42–44]. Short-read RNA-seq often struggles with repetitive regions and long contiguous sequences, leading to fragmented or incomplete transcript assemblies [43,45,46]. As a result, transcript-level regulatory changes across cell differentiation stages remain incompletely characterized.

By contrast, LR sequencing technologies, such as those developed by Oxford Nanopore and PacBio, have revolutionized transcriptomics by producing reads that span entire transcripts, enabling accurate reconstruction of full-length RNA molecules [47,48]. LR sequencing has now been successfully applied to profile the human tissue transcriptome, revealing extensive transcript diversity and previously unannotated isoforms [49]. This breakthrough allows for the identification of novel transcripts and complex splicing events that are often missed by short-read methods [50–53].

In our study, we utilized nanopore RNA-seq to achieve high-quality transcriptome profiling and a comprehensive analysis of DGE, DTE and DTU between human skin-derived fibroblasts, fibroblast-derived iPSC and iPSC-derived iCN. Rather than identifying strictly cell type-specific transcripts, we focused on genes and transcripts that are differentially expressed or used between cell types, thereby capturing transcriptomic transitions along the reprogramming and differentiation trajectory. Our DTU analysis revealed differences in transcript usage between cell types or during cell differentiation, reflecting AS changes potentially influenced by transcriptional, epigenetic or post-transcriptional regulatory mechanisms. Notably, we identified genes with significant changes in transcript expression and usage, including medically relevant genes such as *APP*, *KIF2A* and *BSCL2*, which undergo AS. These findings underscore the complexity of transcript regulation and highlight the importance of incorporating transcript-level analyses to fully understand transcriptomic regulation and disease mechanisms.

## 2. Results

### 2.1. Long-read sequencing uncovers transcript diversity and complexity across cell types

**Figure 1.**
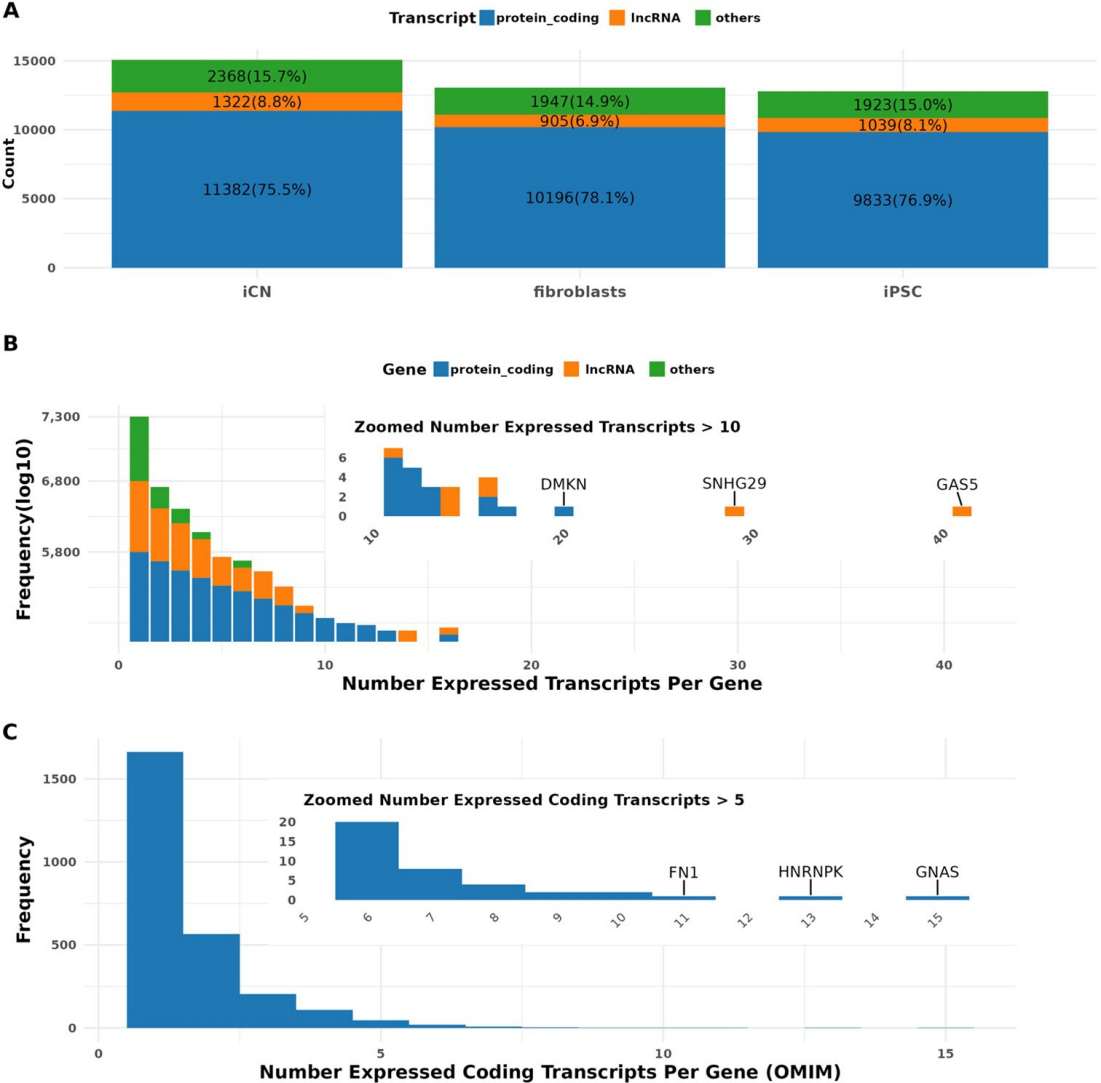
Transcriptome profiling of cultured cells using long-read RNA sequencing. (A) Transcript type distribution per cell type: bar plot showing the number and proportion of expressed transcripts by biotype—protein-coding (blue), long non-coding RNAs (lncRNA, orange) and others (green)—within each cell type (iCN, fibroblasts and iPSC). Expression is defined by a threshold of TPM > 1. This panel reflects total expressed transcript counts in each cell type but does not distinguish whether transcripts are unique to a given cell type or shared across types. (B) Transcript diversity per gene: distribution of the number of expressed transcripts per gene (TPM > 1), aggregated across all cell types. Genes are categorized by transcript biotype. The inset zooms in on genes expressing more than 10 transcript isoforms, including examples such as *DMKN*, *SNHG29* and *GAS5*. Note that this analysis does not resolve transcript diversity by cell type. (C) Protein-coding transcript diversity in OMIM genes: bar plot showing the number of expressed coding transcripts per gene for genes listed in the OMIM database, aggregated across all samples. Most genes express fewer than five isoforms. The inset highlights outlier genes with high isoform diversity (e.g. *FN1*, *HNRNPK*, *GNAS*). As in (B), this plot does not represent cell-type-specific expression but instead summarizes global transcript diversity.

We utilized the Oxford Nanopore Technologies LR sequencing platform to generate an average of 17 722 607 QC-passed reads, enabling us to assess transcript diversity across various cell types and differentiation stages, including human-derived fibroblasts, fibroblast-derived iPSC and iPSC-derived iCN. The properties and purity of cultured iCN were previously shown [54]. Gene and transcript level quantification was performed using our robust pipeline (see §4). A transcript was considered expressed above noise levels if its median TPM exceeded one within each respective cell type, i.e. evaluated independently per cell type. Using this criterion, we identified a total of 21 040 unique transcripts across all three cell types with an average of 15 072 transcripts in iCN, 13 048 in fibroblasts and 12 759 in iPSC (figure 1A; electronic supplementary material, figure S1). Additionally, our analysis revealed a significant number of lowly expressed transcripts (TPM between 0.1 and 1), with a count of 20 939 in iCN, 18 896 in fibroblasts, 19 571 in iPSC (electronic supplementary material, figure S2A).

To assess the accuracy of our LR transcriptomic data across cell types, we implemented a comprehensive multi-step validation process. First, we curated a set of marker genes specific to neuron (*n* = 85), fibroblasts (*n* = 132) and iPSC (*n* = 13) from the PanglaoDB database (https://panglaoDB.se/index.html). We examined the expression of these marker genes within our samples (electronic supplementary material, figure S2B). As expected, the expression patterns of marker genes matched the respective cell types. Next, we compared our results with previously published datasets, including two Illumina short-read datasets [55,56] and a nanopore LR dataset [51]. Principal component analysis (PCA) demonstrated that our data clustered distinctly by cell type, with clear separation and alignment with existing datasets (electronic supplementary material, figure S2C), thereby further validating the accuracy of our transcriptomic profiles. Samples grouped primarily by cell type along the first principal component (PC1), reflecting strong concordance in cell type-specific expression patterns across datasets. Notably, our samples aligned closely with their respective counterparts from published studies, supporting the robustness and validity of our data integration approach.

Residual variation along the second principal component (PC2) was most apparent among iCN and likely reflected biological heterogeneity between donor-derived cell lines. These differences were consistent with known donor- and clone-specific transcriptomic signatures, commonly seen in reprogrammed and differentiated cell models [57,58], and were unlikely to represent technical batch effects, which were effectively minimized during preprocessing.

Transcripts identified in our samples were classified based on the Gencode annotation (v43) into categories such as protein-coding transcripts, long non-coding RNAs (lncRNAs) and other biotypes, including transcripts containing retained introns or subjected to nonsense mediated decay and pseudogene transcripts. On average, 76% of the expressed transcripts in our samples were protein-coding, 8% were lncRNAs, and 15% belonged to other biotypes across all investigated cell types. Specifically, in iCN, 11 382 (75.5%) transcripts were classified as protein-coding, 1322 (8.8%) as lncRNAs and 2368 (15.7%) as other biotypes (figure 1A). These transcript counts were based on a TPM > 1 threshold per cell type and reflected the presence of transcripts in each cell type individually, without distinguishing whether the transcript is cell-type-specific or shared.

To explore isoform diversity per gene, we next examined all transcripts that passed the expression threshold in at least one of the three cell types. Using this approach, we detected 21 040 expressed transcripts from 11 853 unique genes. Interestingly, 60.8% (7201) of these genes expressed only a single transcript (figure 1B). On the other end of the spectrum, three genes demonstrated the expression of more than 20 transcripts each, including two lncRNA genes—*GAS5* (45 transcripts) and *SNHG29* (28 transcripts)—and one protein-coding gene, *DMKN* (20 transcripts) (figure 1B). Additionally, we identified 5135 coding transcripts from 2740 genes defined as medically relevant according to the OMIM database (released at 2023.7). Among these, 1207 (44.5%) genes expressed multiple transcripts (figure 1C). Notably, genes such as the highly complex *GNAS*, which is involved in the assembly of the stimulatory G-protein alpha subunit and possibly involved in imprinting, the novel internal ribosomal entry site-transacting factor *HNRNPK*, and Fibronectin-1 (*FN1*), related to cell growth, differentiation and migration, exhibited over 10 transcripts each (figure 1C). These transcript diversity measurements were computed across all cell types collectively, rather than in a cell-type-resolved manner.

### 2.2. Differential transcript expression reveals key transcripts distinguishing cell types and highlighting disease associations

**Figure 2.**
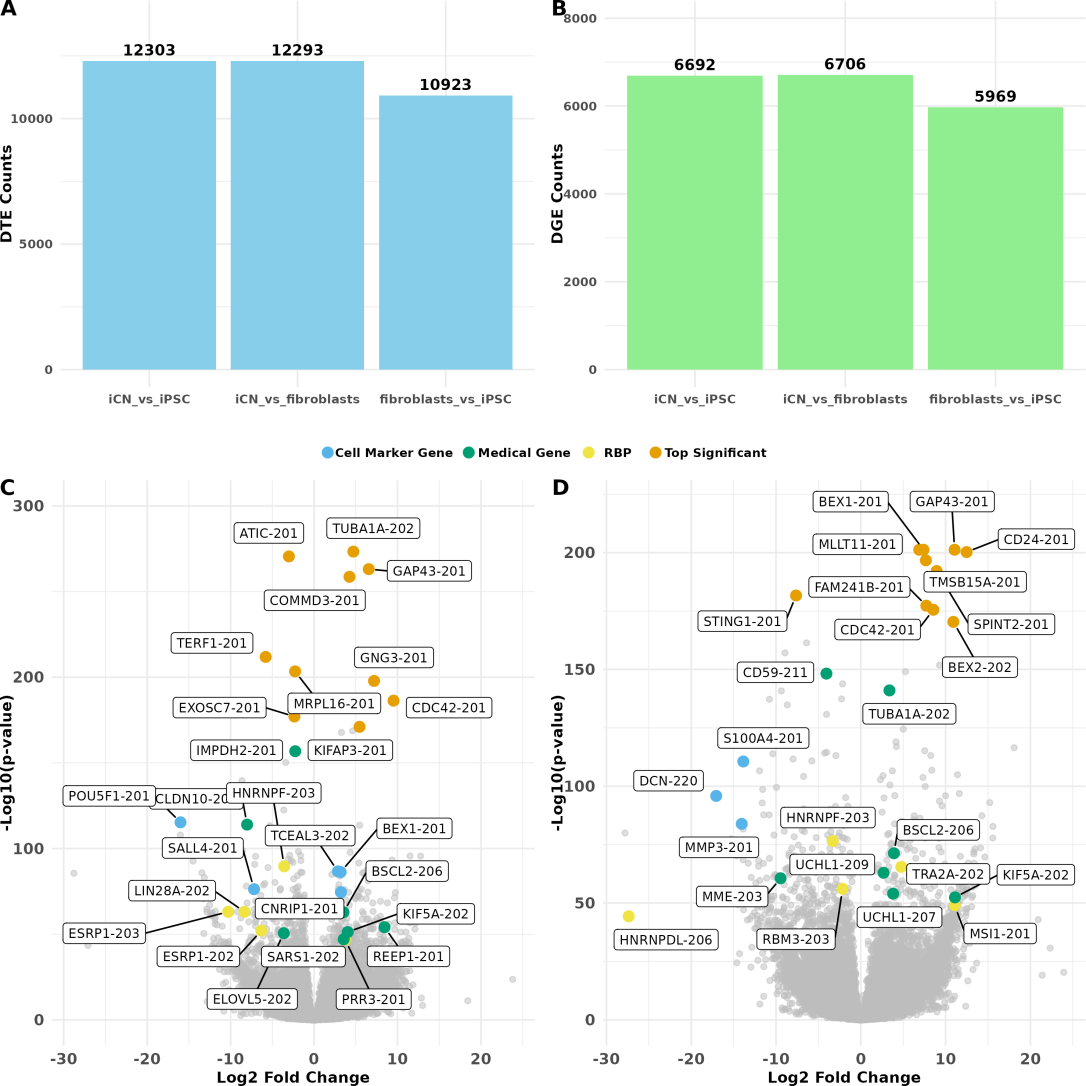
Differential expression analysis. (A) Bar plot showing the number of differentially expressed transcript (DTE, blue) and (B) differentially expressed gene (DGE, green) events in three comparisons: iCN versus iPSC (left), iCN versus fibroblasts (middle), and fibroblasts versus iPSC (right). (C) Volcano plot for DTE of iCN versus iPSC: volcano plot displaying the log_2_ fold change (*x*-axis) against the −log_10_(*p*‐value) (*y*-axis) for each transcript. A set of highlighted significant DTE events (adjusted *p* < 0.05 and |log_2_FC| > 1) are selected and categorized as top high-ranking events by adjusted *p*‐value (orange), originating from medical-associated genes defined by OMIM (green), from RBP mRNAs (yellow), and from cell marker transcripts (blue). (D) Volcano plot for DTE of iCN versus fibroblasts: volcano plot displaying log_2_ fold change (*x*-axis) against the −log_10_(*p*‐value) (*y*-axis) for each transcript. A set of highlighted significant DTE events (adjusted *p* < 0.05 and |log_2_FC| > 1) are selected and categorized as top high-ranking events by *p*‐value (orange), originating from medical-associated genes defined by OMIM (green), from RBP mRNAs (yellow), and from cell marker transcripts (blue). Each comparison group included nine independent replicates (*n* = 9).

Next, we utilized LR RNA-seq data to analyse differential transcript expression (DTE) across various cell types and identified 35 519 DTE events affecting 16 886 unique transcripts across 10 215 genes. Among these comparisons, 12 303 DTE events were identified in the comparison of iCN versus iPSC (Wald test with Benjamini–Hochberg (BH) adjusted *p* < 0.05 and |log_2_FC| > 1), 12 293 events in iCN versus fibroblasts (Wald test with BH adjusted *p* < 0.05 and |log_2_FC| > 1), and 10 923 events in fibroblasts versus iPSC (Wald test with BH adjusted *p* < 0.05 and |log_2_FC| > 1) (figure 2A). The analysis revealed a significant overlap of DTE across the three comparisons. 4262 DTE events were observed in all conditions. By contrast, a smaller subset of DTE transcripts exhibited comparison-specific expression changes. For instance, 988 transcripts were uniquely differentially expressed in the iCN versus iPSC comparison (electronic supplementary material, figure S3A).

Among DTE events detected in the iCN versus iPSC comparison, several transcripts stood out due to their high significance and fold change. These included *TUBA1A*-202, a major component of microtubules crucial for cell structure and intracellular transport [59], *ATIC*-201, which plays an essential role in cellular proliferation [60], and *GAP43*-201, known for its role in neuronal growth, regeneration and synaptic plasticity [61–65]. Additionally, multiple transcripts encoding RBPs were differentially expressed including *HNRNPF*-203, *LIN28A*-202 and *ESRP1*-202/203 (figure 2B). We also observed differential expression of disease-associated genes (e.g. *REEP1*-201, *KIF5A*-202, *EXOSC5*-201), alongside upregulation of neuronal marker transcripts (e.g. *BEX1*-201, *CNRIP1*-201, *TCEAL3*-202) and the expected downregulation of iPSC marker transcripts in iCN (e.g. *POU5F1*-201, *SALL4-*201) (figure 2C).

Similarly, in the iCN versus fibroblasts comparison several transcripts showed significant differential expression (figure 2D). These included *BEX1-201*, known to be involved in neurogenesis and neuronal differentiation [66,67], *GAP43*-201, which plays a crucial role in nervous system development [68,69], and *MLLT11*-201*,* associated with the development and progression of various human cancers [70]. This comparison also revealed changes in transcripts associated with RNA-binding proteins (e.g. *HNRNPF*-203, *RBM3*-203) as well as fibroblast marker genes (e.g. *S100A4*-201, *CD24*-201) (figure 2C). Furthermore, transcripts of disease-associated genes, such as *BSCL2*-206, *REEP1*-201, *UCHL1*-207/209 and *KIF5A*-202*,* all associated with hereditary spastic paraplegia (HSP), were significantly differentially expressed.

The comparison between fibroblasts versus iPSC further highlighted key transcripts associated with cell marker genes (e.g. *S100A4*-201*, POU5F1*-201), RBPs (e.g. *LIN28A*-202) and disease-related genes (e.g. *MME*-203/214*, KIF1A-*207), which may play a role in maintaining fibroblast identity (electronic supplementary material, figure S3B).

In addition to DTE, we investigated differential gene expression (DGE) to provide a broader context for our findings. Importantly, DGE and DTE were conducted as independent, parallel analyses, each using the Wald test with Benjamini–Hochberg adjusted *p* < 0.05 and |log_2_FC| > 1. Our analysis identified 19 367 DGE events across pairwise comparisons between cell types, involving 9200 unique genes. Among these, the comparison of iCN versus fibroblasts exhibited the most DGE events (*n* = 6706), followed by the comparison of iCN versus iPSC (*n* = 6692) and fibroblasts versus iPSC (*n* = 5969). These events were presented side by side to illustrate the relative abundance of significant changes detected at gene and transcript levels (figure 2A,B; electronic supplementary material, figure S4A).

In the DGE analysis of iCN versus iPSC, genes such as *TUBA1A*, *COMMD3*, *ATIC* and *GNG3* exhibited high significance and fold change, mirroring the patterns observed in the DTE analysis. Several other genes, including *REEP1*, *KIF5A*, *LIN28A* and *POU5F1*, were also prominent in both DGE and DTE analyses (electronic supplementary material, figure S4B). Similarly, in the comparisons of iCN versus fibroblasts and fibroblasts versus iPSC, several genes were consistently detected in both DGE and DTE analyses (electronic supplementary material, figure S4C–D). However, some genes, such as *SGCE* and *STRADA* (table 1), did not show significant differential expression at the gene level between cell types but exhibited distinct differential expression patterns at the transcript level (electronic supplementary material, figure S10A,B).

**Table 1.** Summary of representative genes with significant differential transcript usage (DTU) between iCN and iPSC. The table lists key genes, their cellular function, disease association and their regulation status at gene level (upregulated, downregulated or unchanged) in iCN from our DGE analysis.

gene ID	cellular function	disease association	DGE in iCN versus iPSC
MEAF6	transcription regulation and chromatin remodelling [71]	OMIM: *611001	upregulated
BSCL2	lipid storage and metabolism [72]	distal hereditary motor neuropathy and Silver syndrome [73]; hereditary spastic paraplegia type17 [74]; OMIM: *606158, #269700, #608594. #619112, #270685, #615924	upregulated
SEPTIN6	cytokinesis and cellular organization [75,76]	OMIM: *300 683	upregulated
CDC42	cell morphology, migration, endocytosis and cell cycle [77]	cancer and neurodevelopmental disorders [78,79]; Takenouchi–Kosaki syndrome [80]; OMIM: *116952	upregulated
OCIAD1	cell adhesion [81]	OMIM: *300683	upregulated
APP	synapse formation	role in AD [82,83]; OMIM: *104760, #605714	upregulated
PFN2	cell motility and structure [84]	OMIM: *176590	upregulated
C11orf1	male reproduction [85,86]	OMIM: *617615, *600393	upregulated
RTN4	inhibition of nerve regeneration and neural plasticity [87]	OMIM: *604475	upregulated
SGCE	muscle cell membrane stability [88]	muscle integrity and myoclonus-dystonia syndrome [89]; OMIM: *604149, #159900, #608099	not significant
KRT8	cellular structural integrity [90]	OMIM: *1 48 060	not significant
STRADA	mTOR pathway regulation and cell growth [91]	polyhydramnios, epilepsy syndrome [92]; OMIM: *608626, #611087, #234200	not significant
TPD52L2	vesicle-mediated transport and secretion [93]	OMIM: *603747	not significant
KIF2A	intracellular transport, mitotic spindle dynamics [94]	early-onset neurodegeneration [95]; OMIM: *602591, #615411	not significant
LDHA	conversion of pyruvate to lactate [96]	cancer and metabolic disorders [97]; OMIM: *150000, #612933	downregulated
RABGAP1L	intracellular vesicle trafficking [98]	neurodevelopmental syndrome [99]; OMIM: *609238	downregulated
FKBP11	protein folding [100]	OMIM: *610571	downregulated
CMC2	mitochondrial function [101]		downregulated

### 2.3. Differential transcript usage highlights transcript complexity and functional adaptations across cell types

**Figure 3.**
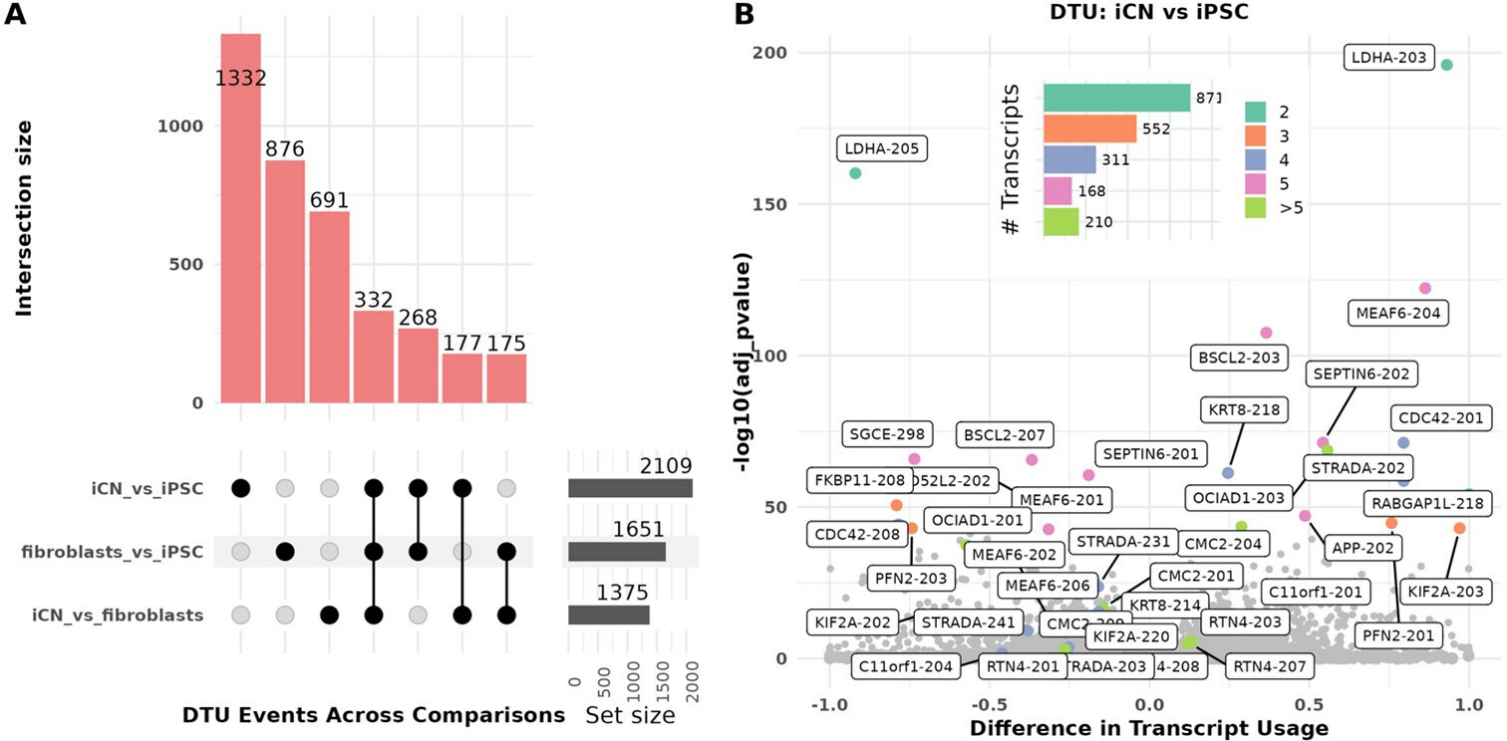
Differential transcript usage (DTU) analysis across cell types. (A) UpSet plot: UpSet plot illustrating the intersection of DTU events across three comparisons: iCN versus iPSC, fibroblasts versus iCN, and fibroblasts versus iPSC. The bars indicate the number of shared and unique DTU transcripts for each comparison. iPSC versus iCN comparison has the highest number of unique DTU transcripts (1332). The bar plot denotes the intersection size, circles denote which comparisons have overlap, and the set size reflects the total number of genes. (B) Volcano plot for iCN versus iPSC comparison: volcano plot showing the difference in transcript usage (*x*-axis) against the −log_10_(*p*‐value) (*y*-axis) for each transcript. Top significant DTUs are highlighted, with notable genes such as *APP*, *KIF2A* and *BSCL2*. Points are coloured based on the number of transcripts per gene across all cell types, as indicated by the inset bar chart (aquamarine = 2 transcripts (*n* = 871); orange = 3 transcripts (*n* = 552); purple = 4 transcripts (*n* = 311); pink = 5 transcripts (*n* = 168); green > 5 transcripts (*n* = 210)). This reflects the overall transcript diversity of each gene, not limited to DTU-involved isoforms. The highest frequency of DTU events occurs in genes with two expressed transcripts, followed by those with three and four transcripts. Each comparison group included nine independent replicates (*n* = 9)*.*

Differential transcript usage (DTU) refers to variations in the relative abundance of transcripts from the same gene across conditions or cell types. These variations often indicate functional adaptations at the transcript level. Genes were considered to exhibit DTU if they had at least one significant DTU event, defined by DRIMSeq-adjusted *p* < 0.05 and an absolute change in isoform fraction (dIF) > 0.1. Among the 4652 genes in our dataset that expressed more than one transcript, we identified a total of 5135 DTU events involving 3851 transcripts from 1894 genes, based on comparisons between cell types. The highest number of DTU events was observed in the comparison of iCN versus iPSC (*n* = 2109), followed by fibroblasts versus iPSC (*n* = 1651), and iCN versus fibroblasts (*n* = 1375) (figure 3A). 1332 DTU events were unique to the iCN versus iPSC comparison, indicating distinct transcript usage patterns in this transition (figure 3A). Interestingly, we observed 332 shared DTU events between all comparisons, which might suggest common regulatory mechanisms (figure 3A).

We next categorized the significant DTU events by AS type and analysed their distribution across the different cell type comparisons. Transition from iPSC to iCN revealed a significant enrichment in iCN for alternative 3′ splice site usage (A3 loss), increased intron retention (IR gain)*,* and mutually exclusive exon (MES) usage (FDR < 0.05) (electronic supplementary material, figure S5). These AS patterns underscore the extent of splicing-mediated transcriptomic remodelling that occurs during cortical neuron differentiation. By contrast, comparisons involving fibroblast exhibited fewer or no significant AS enrichments, suggesting a more limited role for splicing changes in these transitions (electronic supplementary material, figure S5).

To explore the functional relevance of the 1139 DTU genes between iCN versus iPSC, we performed pathway enrichment analysis using Gene Ontology (GO) terms and Reactome pathways. This analysis included all significant DTU genes (*n* = 1139). Enriched biological processes (BP) identified in the GO analysis included regulation of mRNA processing (GO:0050684, intersection size = 24, *p* = 1.88 × 10^−5^), translation (GO:0006412, intersection size = 65, *p* = 0.000201), generation of neurons (GO:0048699, intersection size = 106, *p* = 0.00901), reflecting dynamic post-transcriptional and translational regulation during the transition from iPSC to iCN (electronic supplementary material, figure S6A).

Enriched cellular components (CC) analysis revealed enrichment for focal adhesion (GO:0005925, intersection size = 42, *p* = 0.000525), clathrin coat (GO:0030118, intersection size = 11, *p* = 0.00503), ribosomal subunit (GO:0044391, intersection size = 22, *p* = 0.021), suggesting coordinated regulation of protein synthesis, cytoskeletal remodelling and vesicle trafficking during differentiation (electronic supplementary material, figure S6A).

Enriched molecular functions (MF) included molecular adaptor activity (GO:0060090, intersection size = 105, *p* = 3.74 × 10^−7^), mRNA binding (GO:0003729, intersection size = 39, *p* = 0.00243), cadherin binding (GO:0045296, intersection size = 36, *p* = 0.00363) (electronic supplementary material, figure S6A), highlighting post-transcriptional regulation, protein complex formation, and cell to cell adhesion processes involved in neuronal lineage commitment (electronic supplementary material, figure S6A).

Reactome pathway enrichment analysis further underscored these findings (electronic supplementary material, figure S6B), with significant enrichment in pathways like RNA polymerase II transcription (REAC:R-HSA-73857, intersection size = 120, *p* = 0.000254), metabolism of proteins (REAC:R-HSA-392499, intersection size = 157, *p* = 0.00168), signalling by Rho GTPases (REAC:R-HSA-194315, intersection size = 68, *p* = 0.006), signalling by Rho GTPases, Miro GTPases and RHOBTB3 (REAC:R-HSA-9716542, intersection size = 68, *p* = 0.012), chromatin organization (REAC:R-HSA-4839726, intersection size = 33, *p* = 0.0177), and mitochondrial translation (REAC:R-HSA-5368287, intersection size = 16, *p* = 0.0369). Together, these may reflect the interplay of transcriptional, post-transcriptional and signalling processes that drive neuronal differentiation from iPSC.

Among the 1139 DTU genes identified in the iCN versus iPSC comparison, 525 genes (46%) were OMIM genes, underscoring a potential link between transcript isoform regulation and human disease. An additional 29 of 1139 DTU genes encoded RBPs, pointing to a pivotal role for post-transcriptional regulation during the differentiation of iPSC to iCN. Functional annotation of these disease-associated gene and RBPs revealed clustering in key BPs, including generation of neurons (GO:0048699, *n* = 53), cell division (GO:0051301, *n* = 16), organelle organization (GO:0006996 *n* = 104) and translation (GO:0006412, *n* = 65) (electronic supplementary material, figure S7). These findings suggested that isoform level regulation within disease genes and RBPs may influence essential cellular pathways, thereby affecting both neurodevelopment and disease vulnerability.

Further analysis of DTU event characteristics revealed that genes with two expressed transcripts across all cell types were the most common (*n* = 871) (figure 3B). A similar pattern was observed in the comparison of iCN versus fibroblasts (*n* = 566) and fibroblasts versus iPSC (*n* = 689) (electronic supplementary material, figure S8A–D). Additionally, many genes exhibitng DTU events were found to have more than two expressed transcript isoforms in total (figure 3B). For example, in the iCN versus iPSC comparison, genes such as *RTN4*, *BSCL2* and *APP* showed multiple transcript variants, highlighting the high level of transcript complexity in these genes (figure 3B).

Table 1 presents 18 representative genes, each containing at least one of the top-ranking DTU transcripts (based on adjusted *p*‐value), along with their DGE changes, categorized as upregulated, downregulated or not significant in iCN versus iPSC comparison. The number of transcripts expressed per DTU gene is summarized in the inset bar plot in figure 3B.

To illustrate the diversity of transcript-level regulation among these representative genes, we highlight several examples. *CDC42* displayed differential expression and transcript usage at both the gene and transcript levels (electronic supplementary material, figure S9A). In iCN, *CDC42* was upregulated at the gene level, with transcript *CDC42*-201 predominantly expressed and showing higher usage (electronic supplementary material, figure S9A). By contrast, *CDC42*-208 was the transcript predominantly used in iPSC (electronic supplementary material, figure S9A). *LDHA* was downregulated at the gene level in iCN but exhibited a complex transcript regulation pattern, with some transcripts upregulated and others downregulated, alongside differential transcript usage between iCN and iPSC (electronic supplementary material, figure S9B). Interestingly, other medically relevant DTU genes, such as *SGCE* and *STRADA*, did not show statistically significant changes at the gene level (electronic supplementary material, figure S10A,B). However, both genes exhibited multiple differentially expressed transcripts and notable shifts in transcript usage. For instance, four *STRADA* transcripts (*STRADA*-202, *STRADA*-203, *STRADA-*231 and *STRADA*-241) were identified in our dataset (electronic supplementary material, figure S7B). *STRADA*-202 showed the highest transcript abundance and usage in iCN, while the other three transcripts exhibited higher expression levels and greater usage frequency in iPSC (electronic supplementary material, figure S10B).

**Figure 4.**
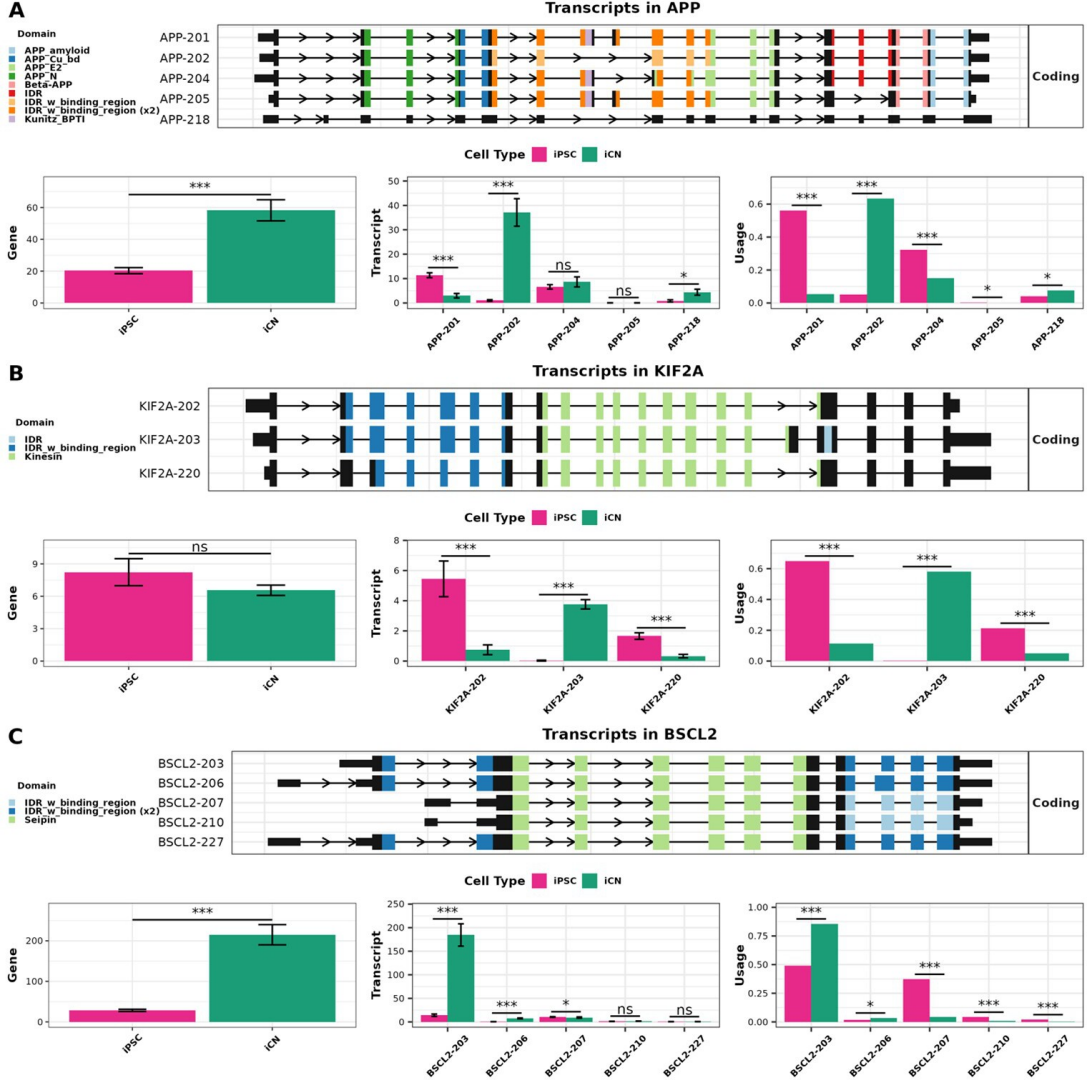
Differential transcript usage analysis for the *APP*, *KIF2A* and *BSCL2* genes. For each of the selected genes (A) *APP* gene; (B) *KIF2A* gene; (C) *BSCL2* gene, the top panel indicates the transcript structures based on Gencode annotation v43, with main protein domains indicated by different colours. Bottom panels: gene expression (normalized TPM) levels (left), transcript expression (normalized TPM) levels (middle), and transcript usage (right) in iPSC (pink) and iCN (green). Differential gene expression (DGE) and differential transcript expression (DTE) were analysed using DESeq2, while differential transcript usage (DTU) was analysed using DRIMSeq. Statistical significance is indicated by ns (not significant), * (*p* < 0.05) and *** (*p* < 0.001). Each comparison group included six independent replicates (*n* = 9) (error bars: ± lfcSE (standard error of the log_2_ fold change) are shown for DGE and DTE plots where applicable; for DTU, error bars are not displayed as DRIMSeq is based on a likelihood ratio framework and does not estimate standard errors or confidence intervals for transcript usage proportions).

To explore the relationship between differential regulation at the gene and transcript levels (DTE, DTU and DGE) in greater detail, we focused on the medically relevant DTU genes *APP*, *KIF2A* and *BSCL2*. It is widely accepted that *APP* is an Alzheimer hallmark gene and plays a significant role as a regulator in neural system development [82]. The alternative splicing of *APP* mRNA can generate approximately 10 different transcripts, which are differentially expressed in various tissues [102,103]. In our analysis, we observed upregulation of *APP* in iCN at gene level (DGE) and identified five distinct *APP* mRNA transcripts (figure 4A). Of these, four (*APP*-201, *APP*-202*, APP*-204 and *APP*-205) are annotated as protein-coding, while one, *APP*-218, is classified as protein-coding, though its coding sequence (CDS) has not been fully defined in the latest Gencode annotation (figure 4A). Our differential transcript analysis revealed that three transcripts (*APP*-201, *APP-*202 and *APP*-218) displayed both differential expression and usage in iCN compared to iPSC (figure 4A). By contrast, *APP*-204 and *APP*-205 exhibited differential usages without differential expressions. To highlight the magnitude of isoform switching, we calculated the values in dIF: *APP*-201 dIF = −0.56, *APP*-202 dIF =+0.48, *APP*-204 dIF = −0.23, *APP*-205 dIF = −0.00075 and *APP*-218 dIF =+0.054. Interestingly, the transcript *APP*-202, also known as *APP*_695_, which lacks the Kunitz-like protease inhibitor (KPI) domain and Ox-2 antigen domain, was predominantly expressed in iCN, while *APP*-201 (*APP*_770_) was primarily expressed in iPSC (figure 4A). This isoform switch towards *APP*_695_ in iCN is consistent with previous findings in Alzheimer’s disease brains, where significant transcript-level changes in *APP* were identified and linked to disease-relevant processing pathways [104].

*KIF2A (*Kinesin Superfamily Protein 2A) plays a crucial role in neuronal migration and differentiation, primarily through its involvement in microtubule dynamics. Mutations in *KIF2A* are associated with cortical dysplasia and early-onset neurodegeneration [95,105,106]. Alternative splicing of *KIF2A* mRNA produces multiple isoforms, each with distinct functional roles. Specifically, in mice, the inclusion or exclusion of exon 18, along with the alternative 5′ splice site selection in exon 5, generates isoforms that differ in their ability to support neuronal migration [107]. In our study, three *KIF2A* transcripts (*KIF2A-202, KIF2A*-203 and *KIF2A-220*) were identified, with significant differential expression and usage between iCN and iPSC (figure 4B). Transcript usage changes were quantified using dIF: *KIF2A*-202 dIF = −0.71, *KIF2A*-203 dIF =+0.97 and *KIF2A*-220 dIF = −0.26. Notably, *KIF2A*-202 and *KIF2A*-220 were predominantly expressed in iPSC, while *KIF2A*-203, an isoform implicated in neuronal migration [107], was more prevalent in iCN (figure 4B). Importantly, despite the differential expression and usage of these transcripts, the overall gene expression of *KIF2A* remained unchanged between iPSC and iCN (figure 4B). This finding suggests that while *KIF2A* is subject to complex regulation at the transcript level, its overall gene expression is tightly controlled, possibly to maintain essential cellular functions.

*BSCL2* (Berardinelli-Seip Congenital Lipodystrophy 2) encodes Seipin, an integral endoplasmic reticulum membrane protein crucial for lipid droplet formation and metabolism. Mutations in *BSCL2* are associated with congenital generalized lipodystrophy type 2, as well as neurodegenerative axonopathies such as hereditary spastic paraplegia and distal hereditary motor neuropathy [108–110]. Aberrant splicing of *BSCL2* has been implicated in several pathological conditions [111]. In our analysis, we detected several *BSCL2* transcripts (*BSCL2*-203, *BSCL2*-206, *BSCL2*-207, *BSCL2*-210 and *BSCL2*-227) with differential expression and usage in iCN compared with iPSC (figure 4C). Transcript usage changes were quantified using dIF: *BSCL2*-203: dIF =+0.365, *BSCL2*-206 dIF =+0.014, *BSCL2*-207 dIF = −0.36, *BSCL2*-210 dIF = −0.038, and *BSCL2*-227 dIF = −0.0198. Notably, *BSCL2*-203 was predominantly expressed in iCN, while *BSCL2*0-207 was more prevalent in iPSC, though expressed at significantly lower levels (figure 4C).

### 2.4. Comparative analysis of DGE, DTE and DTU reveals overlapping and unique gene functional patterns

**Figure 5.**
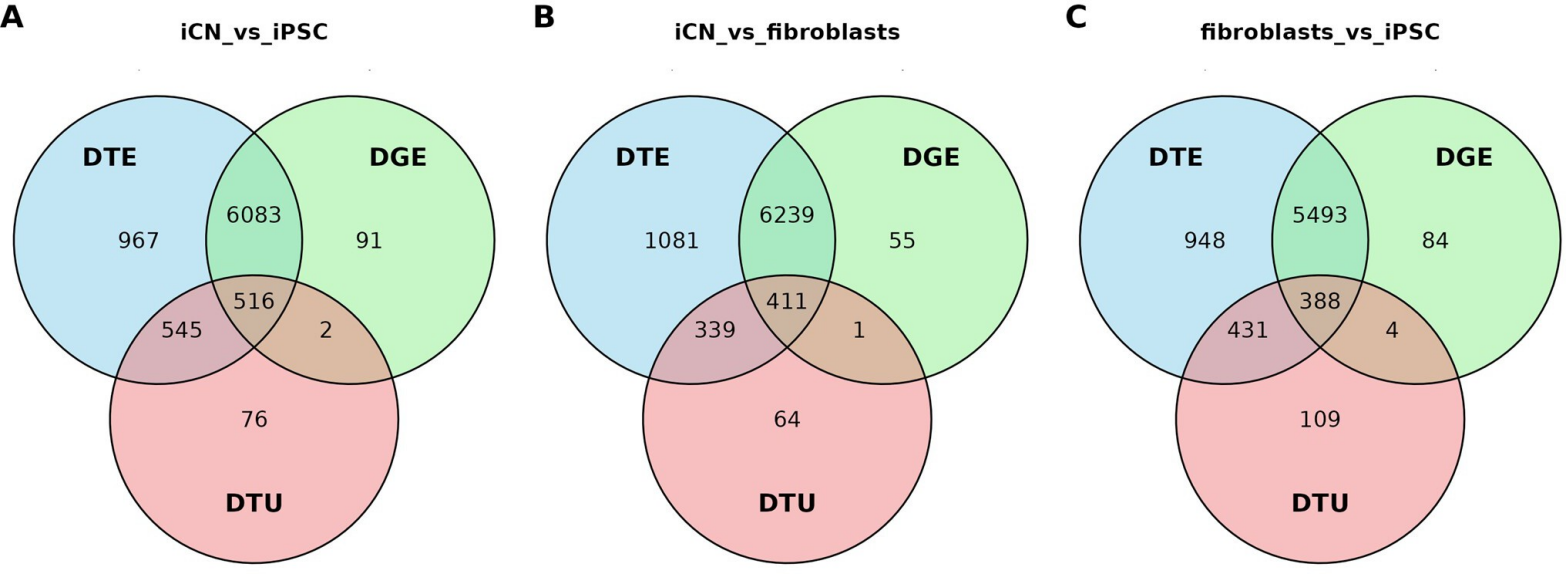
Venn diagrams of differential gene expression (DGE), differential transcript expression (DTE) and differential transcript usage (DTU) across cell type comparisons. Venn diagrams illustrating the overlap between differentially expressed genes (DGE; green), differentially expressed transcripts (DTE; blue), and genes with differential transcript usage (DTU; red) in: (A) iCN versus iPSC, (B) iCN versus fibroblasts, (C) fibroblasts versus iPSC. Each circle represents one of the categories (DTE: top right; DGE: top left; DTU: bottom; red), with the numbers indicating the count of genes in each category and their intersections.

We conducted a direct comparison of the genes identified in each analysis (DGE, DTE and DTU). The greatest overlap was observed between DGE and DTE genes, with a smaller proportion of significant genes identified by all three methods (figure 5A–C).

In the comparison of iCN versus iPSC, 6559 genes were differentially expressed both at the gene and transcript level, with 516 genes shared across all three differential analyses (figure 5A). Similar patterns were observed in the comparisons of iCN versus fibroblasts (figure 5B) and fibroblasts versus iPSC (figure 5C). Interestingly, a notable proportion of DTE genes were not found in the DGE analysis, suggesting that transcript-level regulation plays a pivotal role in cell-specific functions, which may be overlooked by solely investigating gene-level expression. The DTU analysis identified the smallest set of genes, many of which overlap with either DGE or DTE, suggesting that changes in transcript usage often coincide with changes in overall transcript or gene expression levels. The small number of unique DTU genes indicates that few genes exhibit changes in transcript usage without showing changes in transcript abundance or gene expression.

**Figure 6.**
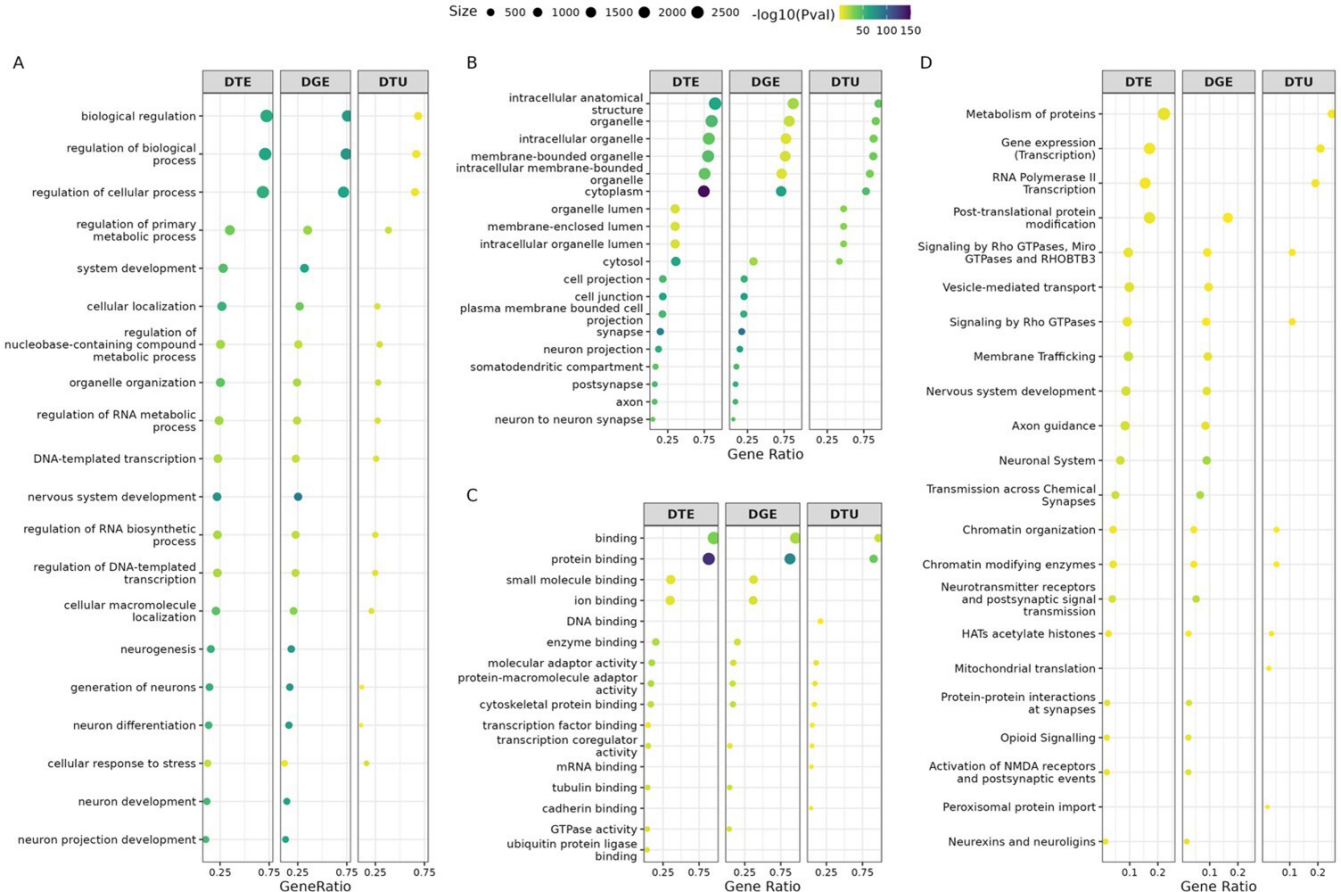
Gene Ontology (GO) term enrichment analysis for DGE, DTE and DTU in iCN versus iPSC. Dot plots illustrate the top 10 terms with the best *p*-values for enrichment from three categories: (A) biological processes (BP), (B) cellular components (CC), and (C) molecular functions (MF) for genes identified through DGE, DTE and DTU analyses. (D) Reactome pathway for genes identified through DGE, DTE and DTU analyses. Each dot represents a specific GO term or Reactome pathway (size of dots indicate the number of associated genes; colour reflecting the −log_10_(*p*‐value), signifying the level of statistical significance).

We performed functional enrichment analysis using gprofiler2 on DGE, DTE and DTU genes across all comparisons. The top 10 GO terms with the best *p*-values of enrichments from each category—BP, CC and MF—are displayed in figure 6 and electronic supplementary material, figures S12–13. In the comparison of iCN versus iPSC, we found significant enrichment of 861 (DGE), 941 (DTE) and 174 (DTU) GO terms (*p* < 0.05). Many of these terms were related to neuronal functions, with ‘neuron’, ‘synapse’ and ‘axon’ prominently appearing in DGE (151 terms, 18%) and DTE (137 terms, 15%), reflecting BPs related to cellular regulation, neurogenesis and neuron development (figure 6A). Additionally, genes identified through DGE and DTE analysis in the iCN versus iPSC comparison were enriched for cellular components of intracellular anatomical structures and organelles (figure 6B). While DGE and DTE sets showed strong enrichments for neuronal functions, the DTU gene set exhibited a distinct pattern. In this case, neuronal pathway enrichment was more limited, with only two significant terms: generation of neurons (GO:0048699) and neuron differentiation (GO:0030182) (figure 6A). Instead, DTU genes were more specifically associated with molecular functions such as to cadherin (GO:0045296), DNA (GO:0003677) and mRNA binding (GO:0003729) (figure 6C), indicating additional layers of transcriptomic regulation that may not be captured by DGE and DTE analyses alone.

To further investigate the unique insights provided by transcript-level analyses, we focused the subset of 1512 genes identified exclusively at the transcript level, i.e. those identified in DTE but not found as to be differentially expressed at the gene level in DGE. These genes, which exhibit transcript-level changes in expression without corresponding gene-level differences, likely reflect regulatory transcript-level regulation that would otherwise be missed in standard gene-level analyses. Notably, this set included 379 OMIM associated genes, 24 RBP genes and 9 genes that overlapped between OMIM and RBP categories, highlighting their clinical and regulatory importance.

To assess their biological roles, we projected these transcript-specific OMIM and RBP genes onto the GO biological process network derived from all DTE genes. The resulting network visualization (electronic supplementary material, figure S10) emphasized the functional involvement of these genes in key biological processes that were not captured by DGE. Highlighted BPs included nervous system development (GO:0007399), RNA splicing regulation (GO:0043484), mitochondrial respiratory chain complex I assembly (GO:0032981) and TORC1 signalling regulation (GO:1903432). Within these pathways, we specifically emphasized genes uniquely detected through transcript-level analyses, demonstrating their critical functional contributions that would be overlooked by gene-level approaches.

In addition to GO enrichment, Reactome pathway analysis further revealed the functional distinctions among these gene sets (figure 6D). While DGE and DTE were predominantly enriched in neurodevelopmental pathways, including axon guidance (REAC:R-HSA-422475), neuronal system (REAC:R-HSA-112316), nervous system development (REAC:R-HSA-9675108) and synaptic transmission (REAC:R-HSA-112314), the DTU gene set exhibited a distinct enrichment profile. DTU genes were associated with pathways related to chromatin organization (REAC: R-HSA-4839726), RNA polymerase II transcription (REAC: R-HSA-73857) and peroxisomal protein import (REAC: R-HSA9033241) pathways. These results suggest that isoform-level regulation plays a key role in fine-tuning gene expression and modulating epigenetic processes, complementing the broader transcriptional patterns captured by DGE and DTE analyses.

A similar enrichment pattern emerged in the comparison of iCN versus fibroblasts, supporting the notion that these regulatory profiles reflect the unique neuronal identity. By contrast, the fibroblast versus iPSC comparison revealed a markedly different set of enriched pathways, primarily associated with RNA/DNA metabolism, cell cycle progression and transcription reflecting regulatory changes characteristic of the reprogramming process rather than neuronal differentiation. Further details on GO term and Reactome pathway enrichment analysis for both iCN versus fibroblasts and fibroblasts versus iPSC are available in electronic supplementary material, figures S12–S13.

In conclusion, the integrative analysis of DGE, DTE and DTU highlighted the complexity of gene regulation across different cellular contexts. While DGE and DTE overlapped substantially, pinpointing to shared biological relevance, DTU offered additional resolution by capturing transcript regulatory events that would otherwise be missed, emphasizing the importance of considering transcript usage in transcriptomic studies.

## 3. Discussion

Our comprehensive transcriptome analysis using nanopore long-read RNA sequencing (LR RNA-seq) has provided valuable insights into the transcript diversity and expression profiles of human iPSC-derived iCN, fibroblasts and iPSC. By integrating DTE and DTU analyses, we uncovered a level of transcriptomic complexity that extends beyond conventional gene expression studies. This approach was essential for identifying transcript-specific regulation and AS events, offering a more nuanced understanding of the functional implications of transcript variation between cell types. The primary goal of our study was to characterize transcript expression and its dynamic changes during cellular differentiation. Importantly, our dataset is intended to serve as a baseline reference for other researchers working with human-derived disease models, providing a valuable framework for comparing transcriptomic profiles and guiding future investigations into disease mechanisms and potential therapeutic strategies as well as choice of appropriate cellular model system.

Our results revealed that iCN exhibited the highest number of expressed transcripts compared with fibroblasts and iPSC. This aligns with findings from Heberle *et al.* [112] and Page *et al.* [49], who also emphasized the expansive transcriptomic landscape required for proper neuronal function [49,112]. This observation is consistent with the complex roles of neurons, which rely on a diverse set of transcripts to support critical processes like synaptic plasticity, neurotransmission and neurogenesis.

Our DTU analysis revealed a cell-type-specific AS pattern, with IR being more prevalent in iCN compared with iPSC. This finding aligns with the emerging view of IR as a regulated and functionally meaningful mechanism during neuronal differentiation [113,114]. Consistent with our findings, Braunschweig *et al*. [113] demonstrated that IR is surprisingly widespread in mammalian transcriptomes and functions as a ‘transcriptome tuning’ mechanism [113]. Their study showed that IR acts through both nonsense-mediated decay (NMD) and nuclear retention, reducing the levels of transcripts that are not required for the physiological identity of a given cell type, especially in the nervous system where IR is more conserved across species. Boutz *et al*. [114] further refined this understanding by describing a distinct class of detained introns (DIs), introns that are retained specifically in the nucleus within polyadenylated transcripts and are not subject to NMD [114]. These DIs are post-transcriptionally spliced in response to cellular signalling, such as Clk kinase activity, suggesting a role for IR beyond transcript suppression, serving as a regulated reservoir of transcripts that can be spliced upon demand to dynamically alter protein production [114]. More recently, Petrić Howe *et al*. [115] identified a class of cytoplasmic intron-retaining transcripts (CIRTs) enriched during motor neuron development [115]. These transcripts were not associated with reduced gene expression but instead showed selective enrichment for RBP and miRNA interactions, suggesting a distinct post-transcriptional regulatory role for IR [115]. Together, these findings suggest that the IR events enriched in iCN represent a multifaceted regulatory mechanism, modulating gene expression during neuronal lineage commitment through both transcript-level suppression and selective post-transcriptional interactions. In our dataset, DTU genes were significantly enriched for functions related to the cytoplasm, protein binding and neuron generation, reinforcing the notion that IR contributes to the functional specialization of the cortical neuron transcriptome.

Within the framework of transcript-level analysis, DTE provides critical insights into how different isoforms of the same gene are expressed under specific conditions, while DTU highlights changes in the relative abundance of transcript isoforms, reflecting regulatory shifts in splicing across cell states or disease contexts. Increasing evidence supports the role of DTE and DTU in the progression of neurodegenerative diseases. For example, in Parkinson’s disease (PD), DTU analysis has revealed 23 splicing events across 19 genes, including *THEM5*, *SLC16A1* and *BCHE*, in the prefrontal cortex, suggesting functional consequences for altered isoform expression [116]. Similarly, in Huntington’s disease (HD), the *HTT* gene produces multiple isoforms via alternative splicing, impacting the aggregation properties of the huntingtin protein and contributing to HD pathology [117].

Building on this evidence, our study underscores the power of DTE and DTU analyses in revealing the functional consequences of AS. For instance, our investigation into the *APP* gene highlighted distinct splicing patterns that are relevant to AD [103]. Alternative splicing of *APP* directly influences the production of amyloid-beta peptides, a hallmark of AD pathology [82,118–120]. Specifically, our combination of DTE and DTU analysis revealed that the neuron-specific isoform *APP_695_* (*APP-*202) was predominantly expressed in iCN, whereas *APP_770_* (*APP-*201) and *APP_751_* (*APP*-204) were more prevalent in iPSC. These findings align with the well-established role of *APP* in AD pathology, where *APP*_695_, lacking the KPI domain, is more susceptible to proteolytic cleavage, leading to the generation of amyloid-β peptides, a hallmark mechanism of AD [121]. The higher expression of KPI-containing isoforms in non-neuronal cells might suggest a protective mechanism against amyloidogenic processing [122–126]. In addition to *APP*, genes such as *BIN1* have been shown to exhibit significant differential transcript expression and usage, particularly in the temporal and frontal lobes of AD patients, further highlighting the pivotal role of AS in neurodegeneration [104]. This observed pattern of isoform expression supports the validity of our transcript-level analyses and highlights the significance of transcript-specific regulation in understanding disease mechanisms.

Moreover, our study demonstrated that transcript differential analyses can uncover cell-type-specific transcript patterns that would otherwise be missed with gene-level analyses alone. For example, we identified key transcript isoforms in *KIF2A* that exhibited distinct splicing patterns during the transition from iPSC to iCN, even though overall gene expression levels showed no significant differences. Specifically, isoforms *KIF2A*-202 and *KIF2A*-220, which lack exon 17, were significantly upregulated according to DTE analysis and predominantly expressed in iPSC according to DTU analysis. By contrast, *KIF2A*-203, which retains exon 17, was upregulated in iCN based on DTE and showed predominant usage according to DTU analysis. This suggests that neurons utilize specific *KIF2A* isoforms during differentiation, likely to support microtubule depolymerization, a process critical for neuronal migration and axonal development [95,127]. Importantly, transcript differential analyses (DTE and DTU) not only identify these transcript patterns but also guides formation of hypotheses on the regulatory mechanisms of AS. For example, the regulation of AS in *KIF2A* may be influenced by neuron-specific RBPs, which are known to bind to intronic sequences and 3′ UTRs, influencing exon inclusion and mRNA stability [128,129]. This mechanism could ensure the inclusion of exon 17 in *KIF2A*-203, preserving its role in microtubule depolymerization, essential for neuronal differentiation and migration [107]. On the other hand, isoforms lacking exon 17, such as *KIF2A*-202 and *KIF2A*-220, may serve broader, more generalized cellular roles.

In the past, genomic mutations were often linked only to gene-level changes, providing limited insight into how these mutations impact specific transcript isoforms. However, our findings demonstrate that while a gene may exhibit straightforward regulation at the gene level, its transcripts can display complex and varied regulatory patterns, as exemplified by *APP* and *KIF2A*. This indicates that transcript-level changes, such as AS or DTU, can lead to the production of isoforms with distinct functional properties, even when gene expression remains unchanged. These transcript-specific variations may drive disease pathology by altering key cellular processes like protein function, localization or molecular interactions. Therefore, transcript-level analyses allow us to directly link genomic mutations to specific transcript isoforms, offering more precise therapeutic targets for intervention. For example, mutations in the *BSCL2* gene, such as the homozygous c.974dupG mutation, have been shown to lead to exon 7 skipping, potentially producing an abnormal transcript [110]. This splicing alteration, which results in the loss of exon 7 in certain isoforms, has been linked to severe neurodegenerative conditions like Celia’s encephalopathy [110]. In our study, we found that the *BSCL2*-203 transcript, which includes exon 7, is the dominant isoform of the Seipin family expressed in iCN. The presence of exon 7 in *BSCL2*-203 may be critical for maintaining normal neuronal function, and any disruption in this splicing pattern, such as the exon 7 skipping caused by the c.974dupG mutation, could lead to pathogenic outcomes. This example illustrates how transcript-specific changes, driven by mutations that cause AS, can be directly tied to disease mechanisms, emphasizing the importance of identifying specific transcripts as potential therapeutic targets. Therefore, one of the most significant contributions of transcriptome differential analyses may be the potential to identify disease-causing targets. In our study, nearly half of the DTU genes in the iPSC-to-iCN transition were linked to OMIM disease loci, and several were annotated as RBPs. Similarly, genes identified exclusively by DTE, not detected by DGE, showed a high proportion of OMIM- and RBP-associated entries. These findings further highlight the clinical and regulatory significance of transcript-level changes, while overall gene expression may remain unchanged, suggesting that AS and isoform shifts may directly contribute to disease mechanisms.

Beyond its biological contributions, this study also provides a valuable benchmark for the transcriptomics field. As bioinformatics tools for LR RNA-seq analysis continue to mature, comprehensive datasets such as ours, spanning fibroblasts, iPSC and induced cortical neurons, can serve as a useful reference for evaluating transcript quantification, alternative splicing detection and isoform usage metrics. We anticipate that this resource will support the refinement of existing pipelines and stimulate the development of new algorithms optimized for long-read sequencing technologies.

While our study provides important insights into transcript-level regulation during neuronal differentiation, several limitations should be acknowledged:

(1) The functional assignment of specific isoforms remains challenging, particularly in the absence of isoform-resolved experimental validation.
(2) Although Oxford Nanopore Technologies LR sequencing enables full-length transcript profiling, it is known to have a higher base-calling error rate compared to short-read platforms, which may affect transcript quantification and splicing accuracy [130–133].
(3) Despite applying batch correction, residual variability across samples, especially in iPSC-derived neurons, may reflect inter-donor and clonal differences, which can be confounding factors in interpretation [58]. These factors should be considered when generalizing our findings, and future work involving patient-derived datasets and orthogonal validation approaches will be necessary to refine isoform-level functional interpretations.
(4) The functional assignment of specific isoforms remains challenging, particularly in the absence of isoform-resolved experimental validation. While we observed transcript usage differences in disease-associated genes such as *KIF2A* and *BSCL2*, the corresponding isoforms are annotated in current reference transcriptomes. However, their isoform-level validation in disease-relevant datasets remains limited, underscoring the need for follow-up studies in patient-derived systems.

In conclusion, by leveraging nanopore LR RNA-seq, we were able to explore transcript diversity at a high resolution, providing a comprehensive framework for understanding transcript-specific regulation and its impact on cellular identity. This understanding is critical for advancing research into neurodevelopment and neurodegeneration. Importantly, we show that LR RNA-seq, combined with DTE and DTU analyses, can uncover the complexities of transcript regulation, particularly how AS shapes cellular identity and function. Transcript-level analysis may prove crucial in the future, as genetic testing alone overlooks changes in isoform expression and AS that may contribute to disease pathology. AS or alterations to the predominantly expressed isoforms in disease-relevant tissues may also account for diseases that currently lack a genetic explanation. Furthermore, identification of disease-relevant transcripts may open potential therapeutic avenues, as interventions targeting specific isoforms could restore normal splicing patterns and prevent the production of pathogenic isoforms [134]. Ultimately, transcript-level analyses have the potential to significantly enhance our approach to genetic testing by identifying mutations that lead to aberrant splicing or isoform production. Taken together, these analyses hold significant promise for advancing our understanding of cellular functions, especially in neurodevelopmental and neurodegenerative diseases, where transcriptomic diversity and splicing dysregulation play pivotal roles. Moving forward, a continued focus on transcript-level changes in various disease models will provide deeper insights into the molecular mechanisms of disease progression and may lead to the identification of novel therapeutic targets for intervention.

## 4. Methods

### 4.1. Generation of human cell lines

The study was approved by the Institutional Review Board of the University of Tübingen Medical Faculty at the University Hospital Tübingen, Germany (IRB: 423/2019BO1). All participants gave their written informed consent to study participation. Human skin fibroblasts were obtained from healthy donors as previously described [135]. Fibroblasts were cultured in DMEM high glucose media (Sigma) with 10% FCS (Life Technologies). From these fibroblasts and iPSC were reprogrammed as previously described [136–138], using episomal plasmids (pCXLE-hUL ID: 270776, pCXLE-hSK, ID: 27078, and pCXLE-hOCT3/4, ID: 27076) [139]. The reprogrammed cells were seeded onto Matrigel-coated (1:60 in DMEM, Corning^®^) 6-well plates in fibroblast media. The next day, the medium was supplemented with 2 ng ml^−1^ FGF2 (Peprotech) and 1% P/S (Gibco). On day 3, medium was changed to Essential 8 (E8; in-house) media with 100 µM sodium butyrate (Sigma-Aldrich) and 0.1% P/S and changed every other day. After 3 to 4 weeks, colonies were picked and expanded. Cryo-stocks were obtained using 50% E8 media with 40% KO-SR (Life Technologies), 10% DMSO (Sigma-Aldrich) and 10 μM Y-27632 (Abcam Biochemicals). A PCR Mycoplasma Test (AppliChem) was performed following manufacturer’s recommendation. Reprogrammed iPSC were differentiated into cortical neurons of layers V and VI. The differentiation followed previously established protocols [54,140,141]. Briefly, iPSC were seeded at a density of 3 × 10^5^ cells cm^−2^ on Matrigel-coated plates (Corning^®^), in E8 medium supplemented with 10 µM SB431542 (Sigma-Aldrich) and 500 nM LDN-193189 (Sigma-Aldrich). The cells underwent neural induction over 9 days. Post induction, on day 9, the cells were split at a 1:3 ratio and then cultured in 3N medium with 20 ng ml^−1^ FGF-2 for an additional 2 days. From day 11 to day 26 after induction (DAI 11-26), cells were maintained in 3N medium, with addition of heparin (100 µg ml^−1^) on DAI 13 and 15. Medium changes occurred bi-daily. iCN were split on DAI 26, with supplementation of 10 µM DAPT (Tocris) and 10 µM PD0325901 (Tocris) in DAI 27 and 29. iCN were maintained until DAI 37 and RNA was isolated according to the manufacturer instructions (Qiagen; RNeasy Mini Kit). Cortical neuron identity was previously shown by immunocytochemistry (neuronal markers: β-III-tubulin, TAU; dendritic marker: MAP2; cortical layer V (CTIP2) and VI (TBR1) markers) and RT-qPCR (cortical layer markers: FOXG1 and PAX6; dendritic marker: MAP2; microtubule-associated marker: DXC) at DAI 36 [54]. A total of four cell lines were used for the experiments (table 2). All cells described were maintained at 37°C and 5% CO_2_.

**Table 2.** Cell lines used for long-read RNA-sequencing experiments.

human-derived fibroblasts	fibroblast-derived induced pluripotent stem cells	iPSC-derived cortical neurons
CO-1	CO-1 2.1	CO-1 2.1
—	CO-2 1.1	CO-2 1.1
CO-3	CO-3 B.1	CO-3 B.1
CO-4	—	—

### 4.2. Long-read RNA-sequencing processing and quality control

RNA concentration was estimated using the Qubit Fluorometric Quantitation and RNA Broad-Range Assay (Thermo Fisher Scientific) and RNA Integrity Number RIN using a Fragment Analyzer 5300 and a Fragment Analyzer RNA kit (Agilent Technologies). Libraries were prepared using a PCR-cDNA Barcoding Kit (SQK-PCB111.24) from Oxford Nanopore Technologies according to manufacturer’s instructions. A total of 200 ng of total RNA was annealed for strand-switching reaction and reverse transcription with Maxima H Minus RT (Thermo Fisher Scientific). The resulting cDNA was amplified with LongAmp Hot Start Taq Master Mix (NEB) according to the protocol from ONT; with following modifications; extension for 9 min with 12 cycles of amplification and a final extension of 10 min. Library molarity was determined by measuring the library size using a FemtoPulse instrument and a Genomic DNA 165 kb Kit (Agilent Technologies) and the library concentration using Qubit Fluorometric Quantitation and dsDNA High sensitivity assay (Thermo Fisher Scientific). An equimolar pool of four barcoded libraries was cleaned up with 0.8 × AMPure XP beads (Beckman Coulter). The pools were quantified and assessed with Qubit and Femto, as individual libraries. The Rapid Adaptors were added to the pool of amplified cDNA and 20 fmol of the library was loaded on a R.9.4.1 PromethION flow cell (FLO-PRO002) and ran on a PromethION instrument in High Accuracy Basecalling mode for 72 h.

Demultiplexing was performed by MinKNOW software. Initially, quality was assessed using the pycoQC tool [142]. Reads were processed with Pychopper (https://github.com/nanoporetech/pychopper). The FASTQ files were mapped to the reference human genome (GRCh38) using the minimap2 tool with the splicing option and quantified using Bambu v3.2.4 [143] to obtain transcript-level counts and CPM. Then CPM was converted to TPM (transcripts per million) with Gencode annotation v43. For each cell type, median transcript expression was calculated across replicates. Transcripts with a median TPM > 1 were defined as expressed in that cell type. Finally, the expressed transcript sets from each cell type were combined to generate a unified list of expressed transcripts across all conditions.

To obtain gene-level expression, we aggregated the TPM values of all expressed transcripts that mapped to the same gene by summing the transcript-level TPMs. These gene-level TPMs were then used for downstream analyses, including differential gene expression.

Principal component analysis (PCA) was performed on three published datasets, along with our own dataset, using the prcomp function in R version 4.3.1. Prior to PCA, the curated published datasets were converted to gene expression TPM values and integrated with our gene expression data. To remove technical batch effects, we applied the removeBatchEffect function from the limma package (version 3.58.1). We then compared the first two principal components of the integrated datasets to assess whether they exhibit similar transcriptome profiles.

### 4.3. Differential expression analysis

Counts from expressed transcripts across all cell types were used to perform differential gene expression (DGE) and differential transcript expression (DTE) analysis using the DESeq2 (version-1.42.1) R package. Pairwise differential tests were conducted between cell types, such as fibroblasts versus iPSC, iCN versus iPSC, and iCN versus fibroblasts. Genes and transcripts were considered to have differential expressions between cell types if their corresponding test adjusted *p*-values were <0.05 and |log_2_FC| > 1. Upregulated genes and transcripts with significant DGE or DTE events (log_2_FC > 1) were selected for Gene Ontology (GO) term enrichment tests. For example, in the iCN versus iPSC comparison, genes upregulated in iCN were used to represent iCN expression. GO term tests were performed using gProfiler2(v0.2.3) in R and the top 10 GO terms with the best adjusted *p*-values from each category—biological processes (BP), cellular components (CC) and molecular functions (MF)—were selected for visualization. Reactome pathway enrichment was also performed using gProfiler2, and significantly enriched pathways (adjusted *p* < 0.05) were included to further interpret the biological functions associated with differentially expressed genes and transcripts.

### 4.4. Differential transcript usage analysis

Differential transcript usage (DTU) analysis was performed using the IsoformSwitchAnalyzeR v2.2.0 framework in combination with DRIMSeq [144,145]. DRIMSeq conducted the DTU analysis based on transcript usage calculated from TPM values of expressed transcripts. Statistical significance was assessed using a Dirichlet-multinomial model with likelihood ratio tests, providing gene- and transcript-level *p*-values. As the model does not estimate standard errors or confidence intervals for transcript proportions, error bars are not included in usage plots. Instead, statistical significance is visualized by annotating adjusted *p*-values (FDR) in the plots.

We integrated the DRIMSeq results with gene and transcript differential expression results produced by DESeq2 (in §4.3) within IsoformSwitchAnalyzeR framework to predict alternative splicing events and their consequences. Additionally, to ensure robust results, we applied several filtering steps as recommended by IsoformSwitchAnalyzeR. Initially, transcripts were filtered based on a minimum gene expression threshold (gene_condition_1 > 1 and gene_condition_2 > 1) to exclude noise. DTU events were identified using DRIMSeq test results with an adjusted *p* < 0.05 and an absolute usage difference |dIF| > 0.1. Identified DTU genes were then selected for GO term enrichment tests. gProfiler2(v0.2.3) in R and the top 10 GO terms with the best adjusted *p*-values from each category—biological processes (BP), cellular components (CC) and molecular functions (MF)—were selected for visualization.

To predict the functional consequences of differentially utilized transcripts, we analysed coding potential (CPC2) [146], protein domains (Pfam) [147], signal peptides (SignalP 5.0) [148], intrinsically disordered regions (IDR) [149], using external sequence analysis tools including Pfam, CPC2, SignalP-5.0 and IUPred2A [150].

## Data Availability

Supplementary material is available online [151].
